# Spectratyping analysis of the islet-reactive T cell repertoire in diabetic NOD Igμ^null ^mice after polyclonal B cell reconstitution

**DOI:** 10.1186/1479-5876-9-101

**Published:** 2011-07-02

**Authors:** Allen M Vong, Nazila Daneshjou, Patricia Y Norori, Huiming Sheng, Todd A Braciak, Eli E Sercarz, Claudia Raja Gabaglia

**Affiliations:** 1 Laboratory of Vaccine Research, Torrey Pines Institute for Molecular Studies. 3550 General Atomics Court. San Diego, 92121, CA, USA

**Keywords:** NOD, NOD.Igμ^null^, diabetes, immunoscope, T cell receptor, B cells, IL-6

## Abstract

**Background:**

Non Obese Diabetic mice lacking B cells (NOD.Igμ^null ^mice) do not develop diabetes despite their susceptible background. Upon reconstitution of B cells using a chimera approach, animals start developing diabetes at 20 weeks of age.

**Methods:**

We have used the spectratyping technique to follow the T cell receptor (TCR) V beta repertoire of NOD.Igμ^null ^mice following B cell reconstitution. This technique provides an unbiased approach to understand the kinetics of TCR expansion. We have also analyzed the TCR repertoire of reconstituted animals receiving cyclophosphamide treatment and following tissue transplants to identify common aggressive clonotypes.

**Results:**

We found that B cell reconstitution of NOD.Igμ^null ^mice induces a polyclonal TCR repertoire in the pancreas 10 weeks later, gradually diversifying to encompass most BV families. Interestingly, these clonotypic BV expansions are mainly confined to the pancreas and are absent from pancreatic lymph nodes or spleens. Cyclophosphamide-induced diabetes at 10 weeks post-B cell reconstitution reorganized the predominant TCR repertoires by removing potential regulatory clonotypes (BV1, BV8 and BV11) and increasing the frequency of others (BV4, BV5S2, BV9, BV16-20). These same clonotypes are more frequently present in neonatal pancreatic transplants under the kidney capsule of B-cell reconstituted diabetic NOD.Igμ^null ^mice, suggesting their higher invasiveness. Phenotypic analysis of the pancreas-infiltrating lymphocytes during diabetes onset in B cell reconstituted animals show a predominance of CD19^+ ^B cells with a B:T lymphocyte ratio of 4:1. In contrast, in other lymphoid organs (pancreatic lymph nodes and spleens) analyzed by FACS, the B:T ratio was 1:1. Lymphocytes infiltrating the pancreas secrete large amounts of IL-6 and are of Th1 phenotype after CD3-CD28 stimulation *in vitro*.

**Conclusions:**

Diabetes in NOD.Igμ^null ^mice appears to be caused by a polyclonal repertoire of T cell accumulation in pancreas without much lymphoid organ involvement and is dependent on the help by B cells.

## Introduction

Type 1 diabetes (T1D) is a T cell mediated disease in which both CD4 and CD8 lymphocytes infiltrate the islets of Langerhans, causing destruction of insulin-producing beta cells and consequently, hyperglycemia. Many characteristics of human T1D are shared with the spontaneous onset of disease in inbred Non Obese Diabetic (NOD) mice, which is commonly used as a model of human pathology. In NOD mice, T cell islet infiltration starts within 3-4 weeks of life, ultimately producing overt diabetes in 80% of female mice beyond 30 weeks of age. Interestingly, NOD.Igμ^null ^mice (which are B cell deficient) do not become diabetic [1], but develop disease if reconstituted with B cells [2]. B cell reconstitution performed early, at 4 weeks of age by a chimera approach (to bypass the MHC class I-mediated rejection), precipitates disease in 65% of the animals starting at 20 weeks of age.

Prior studies have indicated the role of B cells is to stimulate the auto-reactive T cell repertoire by providing enhanced antigen presentation and costimulatory capacities that compensate for natural defects in dendritic cells and macrophage antigen presenting cell populations in NOD mice [3,4]. It is known that to cause disease, the B cells are required to possess the I-A^g7 ^MHC class II molecule [5] and that the specificity of the B cells is also important, as reconstitution of HEL-specific transgenic B cells in NOD.Igμ^null ^mice did not cause diabetes [6]. B cell reconstitution has been shown to restore an autoimmune T cell response to GAD65, an autoantigen in diabetes, we and others have found to be important in disease etiology [2,7]. Importantly, NOD.Igμ^null ^mice have been shown to contain a functional autoimmune T cell repertoire (in the absence of B cells) capable of causing diabetes if transferred into NOD.*scid *mice [8].

CDR3 spectratyping or immunoscope analysis is a highly sensitive technique allowing a non-biased identification of the T cell receptor (TCR) repertoire *ex-vivo *in target organs, spleen and lymph nodes. Diversity in the TCR repertoire is the result of random combinations of V, D and J segments and nucleotide insertions during recombination. This process results in CDR3 lengths being generated that are between four and 14 amino acid residues long. If no T cell expansion is induced within a particular BV family, a Gaussian distribution of CDR3 length is observed, typical of background and polyclonal responses.

In this study, we performed TCR spectratype analysis of V beta (BV) gene expansions at the BV-C beta level on NOD.Igμ^null ^mice in comparison to B cell-reconstituted NOD.Igμ^null ^animals, at different time points post-reconstitution. This allowed us to identify the expanding TCR repertoire infiltrating the islets of NOD.Igμ^null ^mice that are dependent on B cells. We observed that without B cell reconstitution, NOD.Igμ^null ^mice had no pancreatic T cell expansion. No T cell receptor PCR product across the entire BV family repertoire was detected, despite Gaussian BV distributions (non-expanded T cells) being observed in pancreatic lymph nodes and splenocytes of these animals. However, upon B cell reconstitution, a progressive infiltration and increase in diversity of the T cell repertoire was detected in the pancreases, with most of the BV families present at pre-diabetic and diabetic stages. A similar expansion profile of the BV TCR repertoire was also observed in the pancreas of B cell-reconstituted animals treated with cyclophosphamide (CYP). CYP treatment produced accelerated diabetes onset, but no disease in age-matched unreconstituted NOD.Igμ^null ^mice. These results demonstrate that B cells are required for the generation of a pathogenic repertoire of T cells infiltrating the pancreas that promote diabetes.

## Materials and methods

### NOD.Igμ^null ^mouse B cell chimeras and blood glucose measurements

NOD.Igμ^null ^mice (kindly provided by Dr. Serreze, Jackson Laboratories-Bar Harbor, ME) were bred in the TPIMS animal facility. All experiments were performed under approved TPIMS guidelines for animal care and use. B cell reconstitution of NOD.Igμ^null ^mice was performed according to the previously described protocol of Serreze et al [2]. Briefly, 4 weeks old female NOD.Igμ^null ^mice were sub-lethally irradiated (1200 rads) prior to i.v. injection with 5 × 10^6 ^cells from syngeneic age-matched bone marrow (NOD.Igμ^null^) and 3 × 10^6 ^purified B cells from spleens of 4 weeks old NOD mice. Control animals received only NOD.Igμ^null ^syngeneic bone marrow transplant. Animals were grouped at 4 or 5 per cage and blood glucose levels (Accu-Check Compact Plus, Roche Diagnostics) were determined weekly, starting at 10 weeks post B cell reconstitution. Three consecutive blood glucose measurements over 200 mg/dl were the criteria used as positive determination of diabetes.

### Spectratyping analysis

Tissues were processed from animals at different time points of disease from whole pancreata, pancreatic lymph nodes and spleen and spectratyped according to the protocol of Pannetier et al [9]. Total RNA was isolated from pancreatic tissue or cells isolated from spleen or lymph nodes, with a Qiagen RNeasy kit (Hilden, Germany). cDNA was generated by reverse-transcription using an oligo-dT primer ((dT)15) and amplified by PCR using a sense primer for each BV segment and an anti-sense primer (Cbeta145) from the constant region of the beta chain. The generated PCR products were denatured in formamide at 92°C and subjected to analysis on an ABI PRISM 3100 Genetic Analyzer using GeneMapper v4.0 software (Applied Biosystems, Foster City, CA). Lengths for each fragment were determined using the Genescan 400HD ROX size standard (Applied Biosystems). Non-Gaussian peaks representing T cell clonotype expansions were quantified by dividing the expanded peak area by the total area of the entire BV expansion spectratype profile. Only peaks representing 40% or higher of the total profile area were considered significant expansions in our analysis. When 2 expansions were present, the area of each peak needed to represent over 30% of the total area in our analysis, to be considered significant.

### Pancreatic lymphocyte isolation

Pancreatic lymphocytes were isolated as previously described [10]. Briefly, after performing total animal body perfusion with 30 ml of PBS, pancreata were harvested and cut in small pieces in cold high glucose PBS supplemented with 5% fetal bovine serum and the trypsin inhibitors, Aprotinin (Sigma) and TCLK (Sigma). Pancreata were then further digested in warm PBS with Liberase (Roche) for 20 min at 37°C under gentle agitation and lymphocytes isolated by ficoll gradient before characterization of surface markers and phenotypic studies by flow cytometry.

### Flow cytometry and phenotypic studies

In flow cytometry, fluorochrome labeled CD3, CD4, CD8, CD19 and CD44 (supplied by BDSciences, San Diego, CA) were used for analysis. For the phenotypic characterization of cytokine production, *in vitro *stimulation of lymphocytes isolated from pancreas with anti-CD3 and anti-CD28 beads (Invitrogen Dynabeads) was performed, and 5 day supernatants were analyzed for cytokine content by flow cytometry using the CBA kit screening for IL-2, IL-4, IL-6, IL-10, IL-17, IFNg and TNFa (BD Biosciences Th1/Th2/Th17 CBA Kit).

### Cyclophosphamide depletion of regulatory T cells

Regulatory T cells were depleted by using a 200 mg/kg dose of cyclophosphamide as previously described [11]. Briefly, 200 μl of a 20 mg/ml saline solution containing cyclophosphamide (Cytoxan, Mead Johnson, Princeton, NJ) was administered i.p. to 14 weeks old NOD.Igμ^null ^mice that had been reconstituted with B cells. NOD and unreconstituted age-matched NOD.Igμ^null ^animals were used as controls. Cyclophosphamide treatment causes depletion of regulatory T cells in the pancreas for up to 9 days following treatment [11].

### Neonatal NOD.scid transplant under the kidney capsule

Diabetic NOD.Igμ^null ^mice reconstituted with B cells were kept alive by subcutaneous insertion of insulin pellets (Linplant, Linshin, Scarborough, Canada) for 2-4 weeks prior to receiving neonatal (24 hours old) pancreas transplanted under their kidney capsules. Animals were sacrificed 40 hours later and the implants were processed for spectratyping analysis as described above.

## Results

### Profiles of T1D in NOD.Igμ^null ^mice reconstituted with NOD splenic B cells

We studied the progression of diabetes in > 100 NOD.Igμ^null ^mice reconstituted with NOD splenic B cells in comparison to controls (mice receiving NOD.Igμ^null ^bone marrow only and naive unreconstituted NOD.Igμ^null ^animals). In our facilities, we found a 65% incidence of diabetes among the B cell-reconstituted animals, similar to that observed by other groups using this model [2]. In NOD.Igμ^null ^B cell reconstituted animals, the typical time frame for diabetes onset occurred between 18 to 22 weeks. In some mice disease occurred as early as 14 weeks and as late as 34 weeks post-reconstitution (data not shown). Naïve unreconstituted NOD.Igμ^null ^mice or controls (NOD.Igμ^null ^mice receiving bone marrow only) did not develop disease up to 34 weeks of age. However, 10% of these mice kept for long-term observation did develop diabetes very late in life, beyond 12 months of age! Therefore, onset of T1D following B cell reconstitution was roughly equivalent to that as seen for spontaneous disease in the NOD founder strain. A slight delay in disease onset (4 weeks) is found in B cell reconstituted mice. Lack of disease in controls clearly indicated a key role for B cells in the onset of pathology.

### Phenotypic analysis of lymphocyte infiltrate in the pancreata of B cell-reconstituted NOD.Igμ^null ^mice

**Figure 1:**
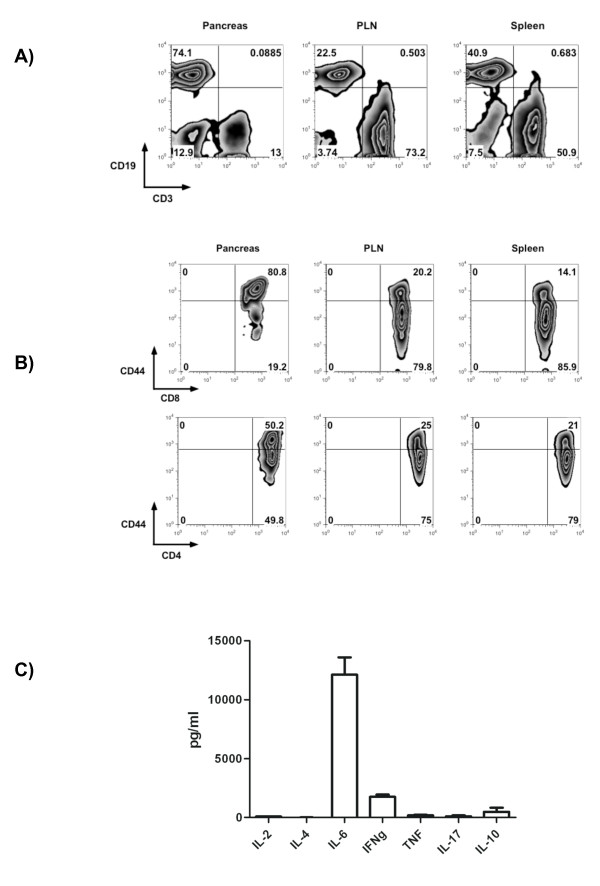
**B and Th1 memory lymphocytes accumulate in the pancreas of NOD.Igμ^null ^mice following B cell reconstitution**. **A) **Flow cytometry analyses of pancreas infiltrating lymphocytes in diabetic animals (between 20 and 30 weeks post-reconstitution) demonstrated an accumulation of CD19^+ ^B cells (74%). T cells accumulate preferentially in pancreatic nodes (73%) and spleen (50%). Data represent 1 of 3 separate experiments with 2-4 animals per group. **B) **The majority of T cells found infiltrating the pancreas expressed memory marker CD44^high ^(80% of CD8^+ ^and 50% of CD4^+ ^respectively). **C) **Pancreas infiltrating lymphocytes were *in vitro *stimulated with anti-CD3/CD28 beads and 5-day supernatants were screened by cytokine bead assays (average and SD of 3 animals).

Flow cytometry was performed in lymphocytes isolated from the pancreas by enzyme digestion and ficoll isolation [10]. Because of the low yield of lymphocytes recovered by the isolation technique in younger animals (9 weeks post-reconstitution), only diabetic mice (between 20 and 30 weeks post-reconstitution) were used for flow cytometric analysis of pancreatic infiltrating lymphocytes. Interestingly, we found that CD19^+ ^B cells represented the majority of cells infiltrating the pancreases representing 64 to 74% of total lymphocytic infiltrate. Only 13-20% of the cells detected were CD3^+ ^T cells (Figure 1A). Amongst the CD3^+ ^T cell compartment, the composition of the CD4^+ ^lymphocytes ranged from 50 to 70%, and CD8^+ ^were 20 to 25%. Approximately half of the CD4^+ ^cells and 80% of CD8^+ ^lymphocytes detected had a memory marker of CD44^high ^expression (Figure 1B). This pattern for the pancreas T cell infiltration was in stark contrast to pancreatic lymph nodes and spleens, where the majority of cells were CD44^low ^(Figure 1B). Interestingly, the B cell accumulation observed in the pancreas was not observed in any other lymphoid organs, including pancreatic lymph nodes and spleen (Figure 1A).

Next, we determined cytokine secretion profile of mononuclear cells infiltrating the pancreas. Lymphocytes isolated from pancreas were *in vitro *stimulated with anti-CD3 and anti-CD28 beads for 5 days. Cytokine production was evaluated by flow cytometry using cytokine bead assays. Upon CD3 and CD28 stimulation, high levels of IL-6 cytokine (12,124 pg/ml) were followed by IFNg (1,757 pg/ml). Low levels of IL-10 (483 pg/ml), TNFa (163 pg/ml), IL-17 (92 pg/ml) and IL-2 (77 pg/ml) were also detected, while IL-4 (0.29 pg/ml) was just above limits of detection (Figure 1C). The observed pattern of cytokine production is characteristic of a Th1 response associated with diabetogenic T cells. This T cell response is likely the consequence of the predominance of B cells activating effector T cells infiltrating the pancreas.

### Significant pancreatic TCR expansions are dependent upon B cell reconstitution in NOD.Igμ^null ^mice

**Figure 2:**
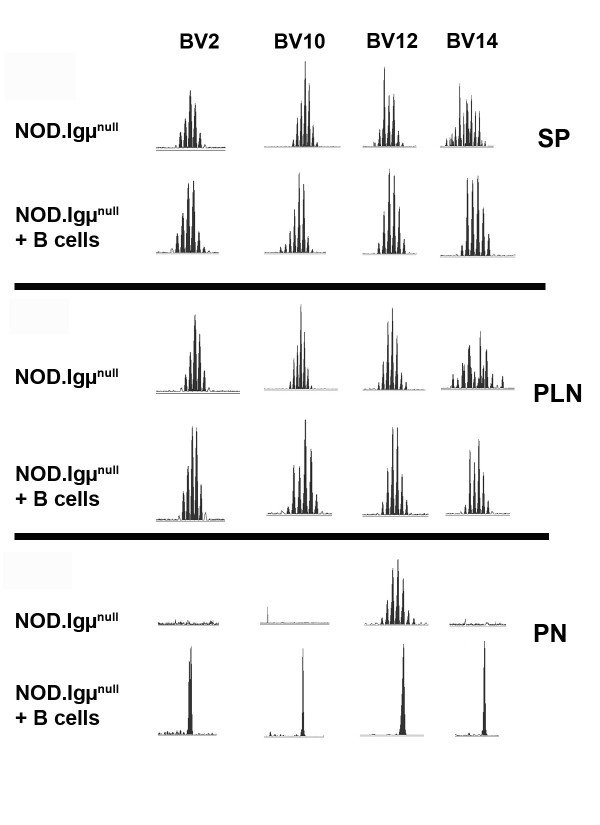
**Representative comparative spectratype analysis for BV families found in spleens, pancreatic lymph nodes and pancreas from untreated and B-cell reconstituted NOD.Igμ^null ^mice**. Splenocytes (SP) and Pancreatic lymph nodes (PLN) were analyzed from naïve NOD.Igμ^null ^and B cell-reconstituted mice (NOD.Igμ^null ^+ B cells). Gaussian profiles for BV2, BV10, BV12 and BV14 families were found in spleens and lymph nodes. Pancreata (PN) of naïve NOD.Igμ^null ^animals had no expansions for these clonotypes, but non-gaussian expansions were detected in high frequency following B cell reconstitution.

Because of the already described role of T cells causing T1D, pancreata from NOD.Igμ^null ^mice were used for spectratyping analysis and detection of T cell receptor V beta chain (BV) expansions at different time points between 5 to 22 weeks of age. Spleen and pancreatic lymph nodes isolated from the majority of NOD.Igμ^null ^mice presented only Gaussian distributions across every BV family tested. An example profile is demonstrated in Figure 2 for BV2, 10, 12, 14 in 14 week-old NOD.Igμ^null ^mice (**PLN**-pancreatic lymph nodes and **SP**-spleens). In the majority of the unreconstituted NOD.Igμ^null ^animals, pancreatic tissue did not generate any detectable PCR product for most BV-TCR families, or presented rare Gaussian expansions (Figure 2). These results indicated that T cells had not infiltrated or were not clonally expanded in the pancreas in the absence of B cells. In contrast, clonotypic expansions were observed in the pancreases of NOD.Igμ^null ^mice at 10 weeks after B cell-reconstitution, indicating a role for B cells in the recruitment and expansion of pathogenic T cells (Figure 2).

Predominant TCR expansion peaks were detected by spectratyping in BV2, 10, 12, 14, 18, 19 and 20 in B cell reconstituted NOD.Igμ^null ^mice. Interestingly, this TCR expansion (non-Gaussian BVs) was specific to the pancreas, as pancreatic lymph nodes and spleens from these animals only produced Gaussian distributions for these same BV families. Total cell numbers recovered from pancreatic lymph nodes were unchanged following B cell reconstitution (data not shown) suggesting that the T cell autoimmune response precipitating diabetes do not appear to be expanding in lymphoid organs.

### B cell reconstitution of NOD.Igμ^null ^mice promotes progressive expansion of the TCR repertoire in the pancreas

**Figure 3:**
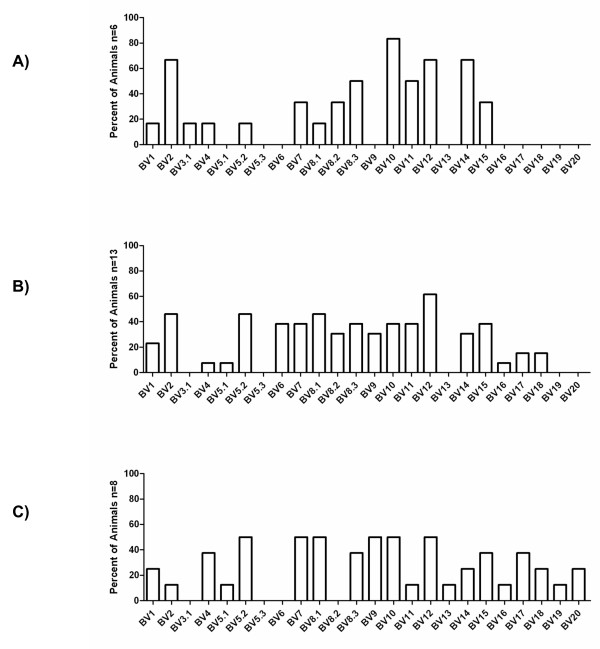
**Policlonal BV repertoire expansions are found in the pancreata following B cell reconstitution in NOD.Igμ^null ^mice**. **A) **Spectratype analysis of BV-BC (Vbeta-Cbeta) expansions for pancreas-infiltrating T cells 10 weeks post-B cell reconstitution demonstrate a polyclonal profile of induced clonotypes, with BV2, BV10, BV12 and BV14 being present on over 60% of the animals, followed next in appearance by BV8S3 and BV11, present in 50% of the mice. **B) **As disease progresses, a higher diversity of clonotypes is observed, particularly for the appearance of BV16, BV17, BV18, BV19 and BV20 in 13-16 weeks post-reconstitution and later **C) **at pre-diabetic stages (19-31 weeks post-reconstitution).

To follow the progression of T cell infiltration after B cell reconstitution of NOD.Igμ^null ^mice, the animals were spectratyped at different time points. At early time points, 9-10 weeks post-B cell reconstitution, the majority of the reconstituted animals accumulated BV2, BV10, 12 and 14 in the pancreas (Figure 3A). At intermediate time points (13-16 weeks post-reconstitution), and even later pre-diabetic and diabetic stages (19-31 weeks post-reconstitution), an increase in the number of BV families was observed. In particular, members of the BV16 to 20 TCR repertoire were present at later time points (Figures 3B and 3C). These results demonstrate that B cell reconstitution is required before a progressive T cell infiltrate is found in the pancreas. The initial TCR repertoire infiltrating the pancreas is less diverse, but ultimately expands over time during diabetogenesis to include a much broader TCR repertoire. This finding is consistent with the spreading and diversification of the pathogenic T cell repertoire [12,13].

### B cell reconstituted NOD.Igμ^null ^mice develop accelerated diabetes following cyclophosphamide-treatment

**Figure 4:**
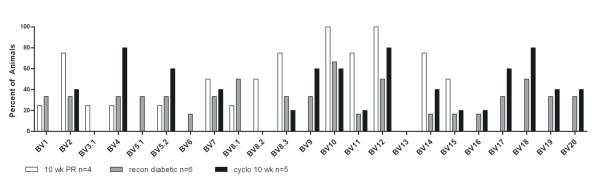
**Spectratyping profile of B cell-reconstituted NOD.Igμ^null ^mice following cyclophosphamide treatment**. Spectratype analysis of BV-BC expansions for pancreas-infiltrating T cells at 10 weeks post-B cell reconstitution of NOD.Igμ^null ^mice are shown, following treatment with cyclophosphamide (black bars) in comparison to 10 weeks old age-matched of NOD.Igμ^null ^mice reconstituted with B cells (white bars), and diabetic NOD.Igμ^null ^mice reconstituted with B cells (grey bars).

To better understand the functionality of the TCR expanded repertoire promoted by B cell reconstitution in NOD.Igμ^null ^mice, we made use of the cyclophosphamide-accelerated diabetes model. Cyclophosphamide (CYP) has been shown to deplete the subset of T cells with regulatory function and accelerate diabetes in NOD mice [11]. We tested whether 14 week-old ureconstituted NOD.Igμ^null ^mice could also develop accelerated disease. Interestingly, we found these animals were resistant to CYP-accelerated diabetes. However, in B-cell reconstituted animals, CYP treatment produced earlier sickness with increased percentages of afflicted animals, compared to age-matched NOD controls (data not shown). We spectratyped the T cell repertoire in the pancreata following CYP-treatment (Figure 4), and found a decrease and/or loss of BV1, BV8 and BV11 TCR expansions. These families are normally present at this time point in B cell reconstituted untreated NOD.Igμ^null ^mice, indicating their potential regulatory function. Furthermore, increased expansions in BV4, BV5.2 and BV9 repertoires were found after CYP treatment, as well as additional expansions of the BV16 to BV20 subsets of T cells, in comparison to age-matched B-cell reconstituted NOD.Igμ^null ^animals. These expansions include BV families directed against antigens proposed as targets of autoimmune response in diabetes pathogenesis [7,14].

### Pancreatic implants into diabetic NOD.Igμ^null ^mice reveal early invasive expansions in select BV clonotypes during tissue rejection

**Figure 5:**
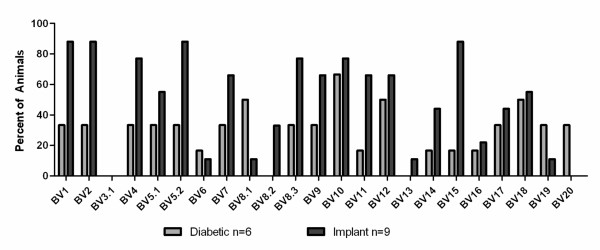
**Spectratyping profile of lymphocytes infiltrating neonatal pancreas implanted under the kidney capsule of diabetic B cell-reconstituted NOD.Igμ^null ^mice**. Spectratyping analysis of BV-BC of neonatal NOD.*scid *pancreas transplanted under the kidney capsule of diabetic NOD.Igμ^null ^mice reconstituted with B cells (black bars). Spectratyping profile of pancreata from diabetic B cell-reconstituted NOD.Igμ^null ^(grey bars).

To search for most aggressive/invasive BV expansions, we studied the pancreatic-graft rejection model. We reasoned that the repertoire potentially mediating early-graft rejection could be as important in initiating T1D. In this model, neonatal NOD.*scid *pancreases were implanted under the kidney capsule of diabetic B cell reconstituted NOD.Igμ^null ^mice. After developing diabetes, mice were kept alive by subcutaneous administration of insulin pellets for 2-4 weeks to stabilize their glycemic levels prior to implantation of neonatal NOD.*scid *pancreas under their kidney capsule. After 40 hours, implants were removed for spectratyping analysis of TCR repertoire of infiltrating T cells. Earlier studies suggested this time point to be the best for examining implant infiltrate, before rejection and fibrosis. Spectratyping analysis of the implants (Figure 5) revealed polyclonal expansions, with several TCR families (BV1, 2, 4, 5.2, 8.3, 10 and 15) being present in most of the implants, including BVs suspected to be of regulatory phenotype based on the cyclophosphamide experiments (Figure 4). Except for BV10, present in similar frequency in the implants and the pancreas, these BV families were found in higher frequencies in the implants, suggesting their higher invasiveness.

## Discussion

Previous studies examining T cell responses and repertoire analysis involved in the autoimmune response of diabetes have produced conflicting results related to the identification of the pathogenic T cell repertoire. Some groups have described polyclonal T cell expansions arising very early in the pancreas being responsible for islet destruction, [15,16], while others have claimed that only particular clonal expansions are the driving force behind autoimmune responses in diabetes [17,18].

These variable findings likely reflect the different techniques employed to characterize T cell responses in the pancreas during the course of spontaneous disease. Here we have employed spectratyping analysis to detect T cell expansions *ex-vivo*, in a non-biased attempt at examining the T cell responses in the pancreas following B cell reconstitution in NOD.Igμ^null ^mice. We found that by 9-10 weeks post-B cell reconstitution, the majority of the animals present pancreatic TCR expansions (at 13 weeks of life). Of note, these animals do not have clonotypic expansions in their pancreatic lymph nodes or spleens, suggesting that clonotypic TCR expansions in lymphoid organs are not involved in disease induction (Figure 2). The initial pancreatic T cell infiltration consisted of several clonotypes, including BV2, BV10, and BV12, clonotypes already described as reactive to insulin or GAD65 [7,14]. BV12 has been found to be enriched in islets of NOD mice when compared to thymus and spleens [15,19]. We also found a BV15 expansion that is a possible candidate for BDC-10.1, a chromogranin A-reactive BV15 T cell [20], which had been previously characterized with a high diabetogenic capacity [14]. As disease progressed, an even larger TCR repertoire infiltrating the organ was observed. This finding is consistent with spreading of the T cell response [21]. Considering the ever-growing list of islet antigens described as being targets of autoimmune response in T1D this polyclonality is expected [17,22]. We found that during the pre-diabetic and diabetic stages, additional expansions in BV16, 17, 18, 19 and 20 families were commonly detected, but these were not predominant in early infiltrates (Figure 3A). It is possible some of these expansions could comprise already described pathogenic clones. A BV16 GAD65-reactive clone (11H11) has been found in the islets of pre-diabetic NOD, with the distinct promiscuous capacity of recognizing different GAD65 peptides using a single TCR [23].

In attempts to find commonalities in clonotype expansions in different pathological states of islet infiltration in the B cell reconstituted NOD.Igμ^null ^model, we also examined the cyclophosphamide-accelerated diabetes and the rejection of pancreatic implants in diabetic NOD.Igμ^null ^B cell reconstituted mice. Following cyclophosphamide treatment, known to eliminate regulatory T cells from the pancreas [11], we found the pancreas-infiltrating repertoire to be quite distinct from that of age-matched non-diabetic NOD.Igμ^null ^B cell reconstituted mice, demonstrating that some regulatory component is also promoted by B cell reconstitution. Interestingly, BV1, BV8 and BV11 T cell expansions were greatly reduced or lost, while a new set of BV4, BV5S2, BV9 and BV16-20 expansions arose, suggesting their role in pathogenicity. Furthermore, BV8S1 and BV8S2 are absent in the cyclophosphamide treated group (a treatment known to destroy regulatory T cells), but present in 50% of the B cell-reconstituted animals. BV8S1 has been previously described as a predominant clonotype infiltrating the islets of partially diabetes-resistant male NOD mice [24] and, interestingly, is also present in the blood of T1D patients [25]. This may indicate that some regulatory component may still be present, although ineffective, at final diseased stages post-B cell reconstitution.

In another approach to address the identity of the pathogenic repertoire, we examined the infiltrating T cells rejecting new pancreatic implants (Figure 5). Pancreatic tissue from neonatal NOD.*scids *transplanted under the kidney capsule of diabetic B cell reconstituted NOD.Igμ^null ^mice were rejected very fast (within 4 days), with the peak of T cell infiltration occurring within 2 days after implantation. The spectratype profile of the BV repertoire from day 2 implants (Figure 5, black bars) was very similar to that seen in the diabetic pancreas (Figure 5, grey bars) but with over 70% of the implants presenting BV1, BV2, BV4, BV5S2, BV8S3, BV10 and BV15 clonotypes. Interestingly, BV1 and BV8 clonotypes were decreased by cyclophosphamide treatment (Figure 4, black bars), therefore, with potential regulatory function. These findings indicate that in B cell reconstituted NOD.Igμ^null ^mice, highly invasive clonotypes predominantly infiltrating transplants are composed of particularly high pathogenic effectors, as well as regulatory T cells.

The breaking of T cell tolerance and passage through "Checkpoint 1-End of Ignorance" [26] by B cell reconstitution, may result owing to two different possibilities. First, homeostatic proliferation of pathogenic T cells following sublethal irradiation, could awaken autoimmune responses. Homeostatic proliferation in an immunodeficient host due to sublethal irradiation or in NOD.*scid *recipients, follows a pattern of expansion that takes circa 6 weeks for complete reconstitution [14]. This mechanism has been shown in the past to generate autoimmune responses [8,27]. Second, autoimmunity could be mediated by the expansion of a T cell repertoire remodeled by the presence of B cells, through their unique antigenic display and enhanced proinflammatory and costimulatory capacities. We argue that homeostatic proliferation seems less likely, as T cells from control animals (reconstituted with bone marrow following irradiation) also go through homeostatic proliferation but do not develop diabetes! B cells from NOD mice are known to produce strong inflammatory responses, when compared to other non-autoimmune strains [28] and present higher levels of costimulatory molecules [29]. Therefore, our data describing a B:T cell ratio of 4 in the pancreases support the second mechanism, and the role for B cells as an important antigen presenting cells in NOD.Igμ^null ^mice. It is likely that B cells help in the induction/activation of the autoreactive TCR repertoire.

During diabetes promoted after B cell reconstitution in NOD.Igμ^null ^mice, B cells encompass over 64% of the lymphocyte population infiltrating the pancreas, despite equal numbers of B and T cells in the other lymphoid organs analyzed (spleens and pancreatic lymph nodes). Interestingly, recent study on the cellularity composition of individual pancreatic islets in female and male NOD mice at different time points of disease evolution do not report a high accumulation of B cells in the pancreas when compared to lymphoid organs [30], while another study identify comparable B:T cell ratios in the spleen for NOD animals [5]. The accumulation of B cells in the pancreas of NOD.Igμ^null ^reconstituted mice could bypass the requirement for T cell lymph node recruitment and may help explain why clonotypic T cell expansions detected in the pancreas are not likewise present in adjacent pancreatic lymph nodes by spectratyping studies. The autoimmune responses may be preferentially localized to the pancreas, induced by larger numbers of antigen presenting B cells (B:T ratio of 4) which could be promoting the effector T cell repertoires unbalancing the regulatory clonotypes. The number of CD19^+ ^B cells circulating in blood post-reconstitution in NOD.Igμ^null ^mice varied from 1 to 13%, with no correlation of higher blood B cells and diabetes onset (data not shown). B cell accumulation in the pancreases but not in lymphoid organs, suggest that the direct activation of effector T cells in the target organ by B cells may be the crucial trigger for disease induction.

B cell accumulation in the pancreas appears to maintain CD4 and CD8 lymphocytes in an activated state (CD44^high ^Figure 1) and IL-6 secreted by the mononuclear pancreatic infiltrate could modulate T cell activity. IL-6 is known to alter phagolysosomal processing, enhancing presentation of cryptic antigenic determinants [31] and to provide survival signal for T cells [32]. Thus, reintroduction of B cells appear to provide an ideal environment for pathogenic T cell activation and survival.

## Conclusions

This study demonstrates that a polyclonal repertoire of pathogenic T cell expansion is dependent upon B cell reconstitution in NOD.Igμ^null ^mice. Diabetes progression appears to be facilitated by B cell accumulation in the pancreas. Interestingly, the clonotypic T cell expansion observed in the pancreas is not observed in other traditionally involved lymphoid organs, including the pancreatic lymph nodes and spleen. The dependence on B cells for the appearance of the pathogenic repertoire of T cells infiltrating the pancreas may help explain why current therapies targeting B cells can affect T1D in NOD mice and humans [33].

## Competing interests

The authors declare that they have no competing interests.

## Authors' contributions

AV, ND and PN performed NOD.Igμ^null ^bone marrow and B cell chimera reconstitutions, blood glucose measurements and spectratype experiments. FACS and cytokine studies were performed by AV, HS and CG. CG, ES and TB conceived and designed experiments. CG and TB wrote the manuscript. Authors have read and approved the manuscript.

## Acknowledgements

This paper is dedicated to the memory of Eli Sercarz, who passed away before the completion of this work. This work was supported by grants to Eli Sercarz: JDRF, Diabetes National Research Group and R01 AI65937-NIH. We are very grateful to Dr. D. Serreze, Jackson Laboratory, for the NOD.Igμ^null ^mice and to Dr. V. Kumar (TPIMS) for critical review of the manuscript.
